# Coping Strategies Among Psychiatric Nurses in a Mental Health Setting in Riyadh, Saudi Arabia: A Cross-Sectional Study

**DOI:** 10.3390/healthcare14183118

**Published:** 2026-09-21

**Authors:** Ahmed Hassan Shujaa, Ahmad M. Rayani

**Affiliations:** 1 Department of Nursing Administration and Education, College of Nursing, King Saud University, Riyadh 11421, Saudi Arabia; 443106295@student.ksu.edu.sa; 2 Community and Psychiatric Mental Health Nursing Department, College of Nursing, King Saud University, Riyadh 12372, Saudi Arabia

**Keywords:** psychiatric nurses, coping strategies, instrumental support, mental health, behavioral disengagement

## Abstract

**Background and Aim:** Psychiatric nurses are frequently exposed to emotionally demanding situations that may increase their risk of occupational stress. Coping strategies are relevant to how psychiatric nurses respond to demanding occupational situations, although their effectiveness and relationships with psychological or professional outcomes were not assessed in this study. This study aimed to assess coping strategies among psychiatric nurses and examine their associations with selected demographic and professional characteristics. **Methods:** A descriptive cross-sectional study was conducted among 225 psychiatric nurses working in Riyadh, Saudi Arabia. Data were collected in person using a structured questionnaire over a period of four months from April to July 2024, comprising demographic and professional characteristics and the Brief COPE inventory. Descriptive statistics, independent-samples t-tests, and one-way analysis of variance (ANOVA) were used to examine coping strategies and their differences according to demographic and professional characteristics. Because multiple comparisons were conducted across coping domains, the Benjamini–Hochberg false-discovery-rate procedure was applied. **Results:** Psychiatric nurses reported the highest mean scores among the assessed coping domains for positive reframing (5.02 ± 1.21), instrumental support (4.68 ± 1.65), active coping (4.62 ± 1.55), and planning (4.62 ± 1.54), based on their mean domain scores. In contrast, behavioral disengagement (2.62 ± 1.69) and humor (2.52 ± 1.30) had the lowest mean domain scores. In the unadjusted analyses, self-blame differed significantly by nationality (*p* = 0.001), with Saudi nurses reporting higher scores (3.65 ± 1.85) than non-Saudi nurses (2.20 ± 2.05). Venting also differed by years of experience (*p* = 0.013), with the highest scores among nurses with <5 years of experience (4.24 ± 1.74). However, after Benjamini–Hochberg false-discovery-rate adjustment for multiple comparisons, neither the difference in self-blame by nationality (adjusted *p* = 0.065) nor the difference in venting by years of experience (adjusted *p* = 0.423) remained statistically significant. No statistically significant differences were observed across coping domains according to gender, educational qualification, or department after adjustment. **Conclusions:** Psychiatric nurses reported greater use of positive reframing, instrumental support, active coping, and planning, while behavioral disengagement and humor had the lowest mean scores. Although unadjusted analyses identified differences in self-blame by nationality and venting years of experience, these differences were no longer statistically significant after adjustment for multiple comparisons. These findings provide descriptive information on coping patterns among psychiatric nurses and may help identify areas for supportive initiatives and future research within psychiatric nursing settings.

## 1. Introduction

Work-related stress is a persistent concern regardless of the profession [1], although nurses are particularly prone to stress because they work in environments characterized by heavy workloads, emotional labor, shift duties, staff shortages, and continuous exposure to suffering, all of which have been associated with poorer psychological well-being and professional functioning [2,3]. Psychiatric nursing involves demanding clinical situations, as psychiatric nurses regularly care for patients with severe mental illness, disturbed behavior, suicidal risk, recurrent relapse, and episodes of aggression or violence, while sustaining therapeutic relationships that require prolonged emotional involvement and may expose them to potentially traumatic and emotionally distressing experiences [4,5]. These demands may place psychiatric nurses at risk of emotional exhaustion, referring to depleted emotional and psychological energy; compassion fatigue, referring to the strain associated with prolonged exposure to the suffering or trauma of others; and burnout, a broader occupational syndrome involving exhaustion, cynicism or depersonalization, and reduced professional efficacy. Together, these concerns highlight the importance of occupational well-being in psychiatric nursing [5,6].

Literature has shown that psychiatric nurses experience work-related stress and depression and use different coping strategies [4,5,6,7]. High turnover intentions, increased absenteeism, and reduced quality of patient care have also been reported among nurses experiencing high levels of occupational stress [8].

Coping is commonly defined as the cognitive and behavioral efforts used to manage internal or external demands that are appraised as taxing or exceeding a person’s resources, a concept widely explained by Lazarus and Folkman’s transactional model of stress and coping [9,10]. This transactional model served as the conceptual framework guiding the present study. According to the model, stress is not determined solely by an event itself but also by how the individual appraises the situation and the resources available to deal with it [10]. In nursing practice, coping responses may involve directly addressing work-related stressors through planning, communication, and help-seeking, whereas other stressors, such as patient suffering, death, aggression, or emotionally traumatic encounters, may require emotional regulation, acceptance, and recovery strategies [4]. Guided by this framework, the present study focused on occupational stress and the coping strategies used by psychiatric nurses in response to their work demands.

Previous research has shown that nurses use a wide range of coping strategies, including seeking social support, problem solving, religious coping, positive reframing, avoidance, emotional suppression, and disengagement, with differences in how these strategies may relate to well-being [4,11,12]. Among nurses in Saudi Arabia, stress and coping have been explored in some clinical areas, and available evidence suggests that occupational stress is common and that coping responses vary according to work setting, experience, and perceived support [11,12]. However, psychiatric nursing differs from many other specialties because its stressors are not limited to workload and staffing demands; they also include managing violent behavior, maintaining therapeutic boundaries, dealing with stigma surrounding mental illness, and coping with the cumulative emotional burden of long-term psychiatric care [4,5].

Evidence from Saudi psychiatric nurses has shown substantial levels of compassion fatigue and has linked professional quality of life to resilience-related factors, suggesting that the ways nurses respond to occupational stress may have important implications for their emotional well-being and sustainability in practice [5,6]. Understanding these coping responses is important because it may help identify strategies that can be supported and responses that may be associated with poorer well-being [2,4,5]. The Saudi healthcare context provides an important setting for examining occupational stress and coping among psychiatric nurses. Cultural values, workplace structures, professional roles, and the organization of mental health services may influence how nurses perceive and respond to occupational stress. However, evidence specifically addressing coping strategies among psychiatric nurses in Saudi Arabia remains limited compared with research from other healthcare settings. Examining these coping patterns in Riyadh may therefore provide context-specific evidence to inform nursing management and staff support initiatives. Differences according to nationality and professional experience may provide useful directions for future research exploring how cultural background and professional experience relate to coping responses among psychiatric nurses. Therefore, this study aimed to assess the coping strategies used by psychiatric nurses and to examine their associations with selected demographic and professional characteristics.

## 2. Methods

A cross-sectional study was conducted among Psychiatric nurses over a period of four months from April to July 2024, using all registered psychiatric nurses who directly participate in patient care in the various clinical units of the Iradah Complex for Mental Health in Riyadh. This research was conducted in accordance with the Declaration of Helsinki guidelines for human research, and participants’ rights and dignity were maintained throughout the research. Before data collection, ethical approval was obtained from the Human Research Ethics Committee at King Saud University (KSU-HE-24-249) on 13 March 2024. In addition, administrative/site permission was obtained from Iradah Complex for Mental Health, Riyadh, through an official letter entitled “Researcher Task Facilitation,” which authorized the researcher to conduct the study and administer the questionnaire to nursing staff at the facility. This site authorization was an administrative permission to facilitate the research activities and did not constitute a separate Research Ethics Committee approval. The study was conducted in accordance with the principles of the Declaration of Helsinki. Before accessing the questionnaire, participants were provided with information about the study and an informed consent statement. Participation was voluntary, and confidentiality and anonymity were assured.

All participants received detailed information before filling out the study questionnaires, explaining the purpose, procedures, the expected time required, the potential risks and benefits, and the measures taken to protect the confidentiality of their information. In addition, there was a statement about participation, which was entirely voluntary, that choosing not to participate would not result in any penalty or loss of benefits, and that participants could withdraw from the study at any time without any consequences. Furthermore, written informed consent was obtained separately from the completed questionnaire to maintain participants’ anonymity. All questionnaire responses were collected anonymously, and no personal identifiers, such as names or employee identification numbers, were recorded. Completed paper questionnaires were securely stored in sealed envelopes to ensure the confidentiality of the data.

### 2.1. Sample Size

The eligible study population comprised approximately 500 nurses at the study setting. The required sample size was calculated using a 95% confidence level, 5% margin of error, and a conservative expected proportion of 50% (*p* = 0.50), which was selected because it provides the maximum required sample size when the underlying population proportion is unknown. For an initial infinite-population sample, the required sample size was calculated as

*n*_0_ = Z^2^
*p* (1 − *p*)/d^2^

where Z = 1.96, *p* = 0.50, and d = 0.05, yielding an initial sample size of 384.16. Because the eligible population was finite (*N* = 500), the finite population correction was applied

*n* = *n*_0_/1 + *n*_0−1/*N*_.

This resulted in a minimum required sample size of approximately 217 participants. To ensure an adequate number of complete responses, recruitment targeted more participants than the minimum required. A total of 250 nurses were approached, of whom 250 responded, giving a 100% response rate. Following data screening, 25 questionnaires were excluded because they did not meet the predefined eligibility/completeness criteria, leaving 225 participants for the final analysis.

### 2.2. Sampling Design

A probability sampling method was applied, and stratified random sampling was used to ensure representation of relevant subgroups within the eligible nursing population. The population was stratified according to professional rank and clinical unit, with gender considered as a secondary stratification variable. These variables were selected because they represented the main organizational and professional characteristics used to classify nurses within the study setting and allowed representation across different professional roles, clinical areas, and genders. The strata were established from the nursing administration roster, which served as the sampling frame [13,14]. Within each stratum, nurses were selected using a computer-generated random number procedure. The number of nurses selected from each stratum was proportional to the size of that stratum in the eligible population, thereby maintaining the relative distribution of nurses across the defined strata. No stratum was intentionally oversampled.

The inclusion criteria include selected individuals who are most likely to offer deep, pertinent, and personal insights on occupational stress issues among psychiatric nurses. The participants should be (a) registered nurses (RNs) actively working at the Iradah Complex for Mental Health. This requirement guarantees that the participants’ experience is relevant and freshly tied to the unique environment of the study [15,16]. Moreover, (b) at least a full-time clinical stint with the complex is required. Having participants who satisfy the one-year clinical experience threshold is crucial, as it enables research participants to endure the initial orientation and transition phases so that their reported stress and coping in the study reflect the realities of the workplace rather than the initial overwhelming period of a new job in a challenging field. (c) The nurse must have a license to practice the profession from the Saudi Commission for Health Specialties (SCHS) and (d) provide informed consent and be willing to complete the full questionnaire. Finally, participants should be actively involved in psychiatric patient care. Exclusion criteria, on the other hand, are in place to protect potential participants and the data. They were excluded if, at the time of data collection, (a) they were currently on long-term leave (medical, maternity, sabbatical), as their estimation of occupational stress at the time would be invalid; (b) they were staff nurse with less than a year of experience in psychiatric nursing; (c) they were nurses not currently employed in a psychiatric setting; (d) they were unwilling or unable to commit to the entire process; or (e) their primary functions were administrative, teaching, or research-related, as the stressors associated are strikingly different from those encountered in direct patient care.

### 2.3. Procedure of Data Collection

The study’s purpose, procedures, benefits, risks, and confidentiality were explained verbally and in writing through the participant information sheet. Interested nurses received a sealed envelope containing the survey, information letter, and consent form. The consent form stated that participation is voluntary, and you can withdraw without penalty. Written informed consent was obtained before participation. All questionnaires were anonymous; no personal details were asked for to encourage honest responses. The data collection was done over 4 months. Reminders from unit managers (who did not know who had replied) were sent after 2 weeks to maximize the response rate. Paper surveys were returned in a sealed box and manually entered into a secure database by the researcher.

### 2.4. Data Collection Instruments

Coping strategies were assessed using the **28-item Brief COPE**, developed by Carver (1997) [17]. The Brief COPE is a self-report instrument designed to assess the coping responses individuals use when dealing with stressful or demanding situations. It comprises 28 items grouped into 14 two-item domains: active coping, planning, positive reframing, acceptance, humor, religion, use of emotional support, use of instrumental support, self-distraction, denial, venting, substance use, behavioral disengagement, and self-blame [17]. Each item is rated on a 4-point response scale ranging from 0 (“I haven’t been doing this at all”) to 3 (“I’ve been doing this a lot”). Each two-item domain is scored by summing its two items, resulting in a possible score range of 0–6, with higher scores indicating greater reported use of the respective coping strategy. The Brief COPE was selected because it assesses a broad range of cognitive and behavioral coping responses and allows individual coping strategies to be examined separately. Its use was also consistent with the Lazarus and Folkman transactional model of stress and coping, which served as the conceptual framework for the study and emphasizes the role of appraisal and coping responses in dealing with demanding situations [18].

All 14 Brief COPE domains were initially included in the questionnaire. However, the two items comprising the substance-use domain received no valid responses from the participating nurses. Consequently, a substance-use domain score could not be calculated, and this domain was excluded from subsequent analyses. The remaining 13 domains (26 items) were analyzed separately, with each domain having a possible score range of 0–6. Higher scores indicated greater reported use of the corresponding coping strategy. No global or overall coping score was used in the analysis. This decision was made because the Brief COPE assesses distinct coping responses and does not provide a universally applicable overall score representing coping effectiveness or adaptiveness. Similarly, no predefined adaptive/maladaptive classification was applied to the coping domains. The present study therefore focused on the reported frequency of individual coping strategies rather than interpreting greater use of a strategy as evidence of greater effectiveness or psychological benefit. No separate instrument was used to measure occupational stress in the present study. The study focused on assessing coping strategies and their associations with selected demographic and professional characteristics. Accordingly, occupational stress was not quantified as a separate study variable, and no statistical analysis was conducted to assess occupational stress as an independent predictor of coping.

### 2.5. Theoretical Framework

The study was conceptually guided by Lazarus and Folkman’s transactional model of stress and coping [9,10]. The model proposes that individuals respond to stressful situations through cognitive appraisal and coping processes, with coping involving cognitive and behavioral efforts to manage demands perceived as challenging or exceeding available resources. In the present study, occupational stress among psychiatric nurses was considered the stressful context, while the coping responses reported through the Brief COPE represented the strategies used to manage these demands. The Brief COPE domains encompass problem-focused responses, such as active coping and planning, emotion-focused responses, such as acceptance and emotional support, and responses that may reflect less constructive coping, such as denial, behavioral disengagement, and self-blame. Consistent with the transactional model, the present study used occupational demands as the contextual background and examined the coping responses reported by psychiatric nurses across the individual Brief COPE domains; the model provided a conceptual framework for understanding the coping responses reported by psychiatric nurses in the context of occupational demands. The Brief COPE domains were examined individually to describe the range and frequency of coping responses and to determine whether these responses differed according to selected demographic and professional characteristics. Because the study was cross-sectional and focused on coping strategies, the findings were interpreted as descriptive associations rather than evidence of causal relationships or coping effectiveness.

### 2.6. Validity and Reliability

The Brief COPE Scale has demonstrated good psychometric properties in previous studies. Reported Cronbach’s alpha coefficients for the subscales typically range from 0.60 to 0.90, indicating acceptable to high internal consistency [17]. A pilot study was conducted before the main data collection to finalize the research instruments, test the procedures, and identify any logistical or methodological problems. The pilot study was conducted among a small sample of 20 psychiatric nurses from the Iradah Complex who met the inclusion criteria. No changes were made to the scale. The objectives of this pilot are threefold. Firstly, it tested the clarity and coherence of every item in the scales; it asked participants if any question was confusing, ambiguous, or if their thinking was incoherent. These were good points for making linguistic adjustments in the instruments. Secondly, pilot data were analyzed to calculate internal consistency reliability, i.e., Cronbach’s alpha for overall scales and their subscales, and compared with the original study and other validation studies. This gave an initial idea of whether the instruments work well in this population or if some subscales need to be modified or deleted. Thirdly, the pilot tested the logistical procedures, including the time to complete the survey, the effectiveness of the distribution and return mechanisms (paper-based), and how easy the participant information sheet and consent procedure are to understand.

### 2.7. Data Analysis

Data were analyzed using IBM SPSS Statistics version 26. Descriptive statistics, including frequencies, percentages, means, and standard deviations, were used to summarize participants’ characteristics and coping scores. The Shapiro–Wilk test was used to assess the normality of continuous variables. Independent-samples t-tests were used to compare mean scores for coping domains between two-category variables, while one-way analysis of variance (ANOVA) was used for variables with more than two categories. Because multiple comparisons were performed across the 13 Brief COPE domains and the demographic and professional characteristics, the Benjamini–Hochberg false-discovery-rate (FDR) procedure was applied to reduce the likelihood of Type I error. Unadjusted and FDR-adjusted *p*-values were reported where applicable. Statistical tests were two-sided, and an unadjusted *p*-value < 0.05 was considered nominally statistically significant; findings were interpreted primarily on the basis of the FDR-adjusted results for the multiple-comparison analyses.

## 3. Results

As presented in Table 1, the characteristics of the participating nurses (*n* = 225) indicate that the majority were Saudi nationals (76.0%), with a fairly even gender distribution (53.8% male, 46.2% female). The majority of nurses fell within the 31–40 age range (55.1%), possessed bachelor’s degrees (64.4%), and had 6–15 years of experience (60%). The participants mostly worked in the inpatient wards (76.0%) and outpatient clinics.

**Figure 1:**
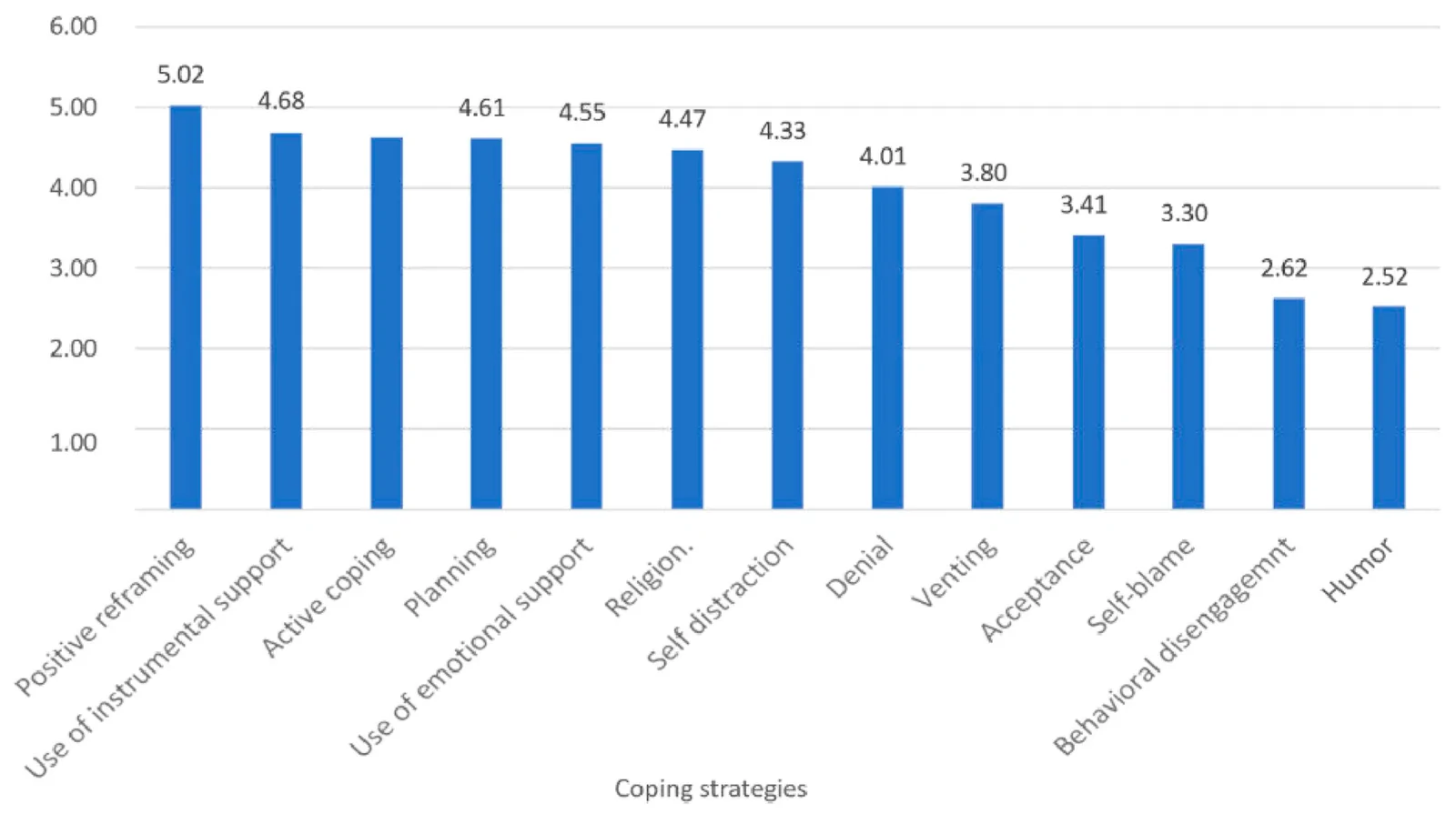
Mean score for the different coping strategies adopted by nurses in response to occupational demands.

As presented in Table 2 and Figure 1, the mean scores for the 13 assessed coping domains ranged from 2.52 to 5.02, with each domain having a possible score range of 0–6. Positive reframing was the most frequently reported coping strategy (M = 5.02, SD = 1.206), followed by instrumental support (M = 4.68, SD = 1.649), active coping (M = 4.62, SD = 1.546), and planning (M = 4.62, SD = 1.540). Other frequently reported coping strategies included emotional support (M = 4.56, SD = 1.677), religion (M = 4.47, SD = 1.544), and self-distraction (M = 4.34, SD = 1.606). Lower mean scores were observed for humor (M = 2.52, SD = 1.303), behavioral disengagement (M = 2.62, SD = 1.692), self-blame (M = 3.30, SD = 1.994), acceptance (M = 3.41, SD = 1.808), and venting (M = 3.80, SD = 1.696). The substance-use domain was not included in the analysis because neither of its two items yielded a valid response in the study sample.

As presented in Table 3, variations in the use of different coping strategies were examined according to the characteristics of the psychiatric nurses. In the unadjusted analyses, two nominally significant differences were identified in the unadjusted analyses at *p* < 0.05: self-blame according to nationality and venting according to years of experience. However, neither remained statistically significant after Benjamini–Hochberg false-discovery-rate adjustment at *p* < 0.05. Self-blame differed significantly according to nationality (*p* = 0.001), with Saudi nurses reporting higher self-blame scores (M = 3.65, SD = 1.852) than non-Saudi nurses (M = 2.20, SD = 2.050). Venting also differed significantly according to years of experience (*p* = 0.013), with nurses having <5 years of experience reporting the highest venting scores (M = 4.24, SD = 1.742), followed by those with 5–9 years (M = 4.18, SD = 1.824), 10–15 years (M = 3.60, SD = 1.462), and >15 years of experience (M = 3.21, SD = 1.833). However, after adjustment for multiple comparisons using the Benjamini–Hochberg false-discovery-rate procedure, neither the difference in self-blame by nationality (adjusted *p* = 0.065) nor the difference in venting by years of experience (adjusted *p* = 0.423) remained statistically significant. No statistically significant differences were observed across the coping domains according to gender, educational qualification, or department (all unadjusted *p* > 0.05).

## 4. Discussion

The present study examined the coping strategies reported by psychiatric nurses working in Riyadh, Saudi Arabia, and explored whether these strategies differed according to selected sociodemographic and professional characteristics. Among the 13 valid Brief COPE domains assessed, positive reframing was the most frequently reported coping strategy, followed by instrumental support, active coping, planning, emotional support, religion, and self-distraction. Behavioral disengagement and humor had the lowest mean scores. Although two statistically significant differences were identified in the unadjusted analyses—self-blame according to nationality and venting according to years of professional experience—neither remained statistically significant after Benjamini–Hochberg false-discovery-rate adjustment. Similarly, no significant differences were observed in coping scores according to gender, nationality, age, educational qualification, department, or years of professional experience. These findings provide a descriptive picture of coping patterns among psychiatric nurses in this mental health setting.

Positive reframing had the highest mean score among the assessed coping domains (M = 5.02 ± 1.21), followed by instrumental support (M = 4.68 ± 1.65), active coping (M = 4.62 ± 1.55), and planning (M = 4.62 ± 1.54). Emotional support (M = 4.56 ± 1.68), religion (M = 4.47 ± 1.54), and self-distraction (M = 4.34 ± 1.61) were also relatively frequently reported. Because each Brief COPE domain has a possible score of 0–6, these findings indicate that participants reported these coping responses more frequently than several of the other assessed strategies.

These findings can be interpreted within the Transactional Model of Stress and Coping proposed by Lazarus and Folkman [18]. From this perspective, coping represents the cognitive and behavioral efforts individuals use to manage stressful demands following their appraisal of a situation. The frequent use of active coping, planning, positive reframing, and social support among psychiatric nurses may indicate frequent use of cognitive, behavioral, and interpersonal coping responses when dealing with occupational demands [9]. Similarly, the use of religion may reflect an additional source of emotional and psychological support within the Saudi cultural context. However, the cross-sectional nature of the study does not allow us to determine whether these coping strategies reduce occupational stress or improve psychological outcomes. The relatively high use of positive reframing may be particularly relevant to psychiatric nursing. Positive reframing involves attempting to interpret a difficult situation from a different or more constructive perspective. Psychiatric nurses may encounter complex clinical situations requiring emotional flexibility, sustained therapeutic communication, and adaptation to patients’ changing behavioral and psychological needs [19]. In such circumstances, cognitive approaches that involve finding alternative perspectives may represent one way of responding to challenging professional experiences. However, the present study did not assess whether positive reframing or any other coping strategy resulted in better psychological or occupational outcomes. Therefore, the finding should be interpreted as a pattern of reported coping rather than evidence of effectiveness.

The relatively high scores for active coping and planning provide further evidence that nurses commonly reported coping responses involving direct engagement with challenging circumstances. Active coping reflects efforts to take action to improve a situation, whereas planning involves considering strategies and determining steps to address a problem. These approaches are conceptually consistent with problem-focused coping described within Lazarus and Folkman’s Transactional Model of Stress and Coping [10,18]. According to this model, coping involves cognitive and behavioral efforts to manage demands that are appraised as challenging or taxing. The present findings suggest that psychiatric nurses reported using both cognitive and behavioral approaches when responding to challenging circumstances in their professional environment.

Instrumental support and emotional support were among the most frequently reported coping strategies. Instrumental support involves obtaining advice, assistance, or practical help from other people, whereas emotional support involves seeking comfort, understanding, or emotional assistance [20,21]. The relatively frequent use of both forms of support may reflect the collaborative nature of psychiatric nursing. Psychiatric care commonly involves interaction with nurses, physicians, psychologists, social workers, and other members of the multidisciplinary team. Professional relationships may therefore provide opportunities for nurses to seek advice, discuss difficult situations, and obtain emotional reassurance.

Previous research has identified social support as an important coping resource among healthcare professionals and nurses [11,12,22]. The present findings are broadly consistent with this literature in showing that both instrumental and emotional support were among the more frequently reported coping responses. Nevertheless, because the present study did not directly assess the availability, quality, or effectiveness of workplace support, the findings cannot establish that organizational support caused or improved nurses’ coping.

Religion was also among the more frequently reported coping strategies (M = 4.47 ± 1.54). Religious coping may involve seeking comfort through religious or spiritual beliefs and engaging in practices such as prayer or meditation. This finding may be particularly relevant in the Saudi Arabian context, where religious and spiritual practices may constitute an important source of meaning and emotional support. Similar use of religious coping has been reported among healthcare professionals in Saudi Arabia [23]. However, the present study did not examine the specific religious practices used by participants or their perceived benefits. Therefore, the finding should be understood as an indication that religious coping was commonly reported rather than as evidence of its effectiveness.

Humor had the lowest mean score (M = 2.52 ± 1.30), followed by behavioral disengagement (M = 2.62 ± 1.69). Self-blame (M = 3.30 ± 1.99), acceptance (M = 3.41 ± 1.81), and venting (M = 3.80 ± 1.70) were also less frequently reported than positive reframing, support-seeking, active coping, and planning. The relatively low reporting of behavioral disengagement may indicate that participants were less likely to report withdrawing efforts to deal with challenging circumstances. Similarly, the lower frequency of humor may reflect the nature of the clinical environment and the types of coping responses that participants considered appropriate in their professional context. However, these findings should not be interpreted as demonstrating that these strategies are inherently ineffective or harmful. The Brief COPE measures different coping responses rather than determining whether a particular response is universally beneficial or detrimental. The appropriateness and consequences of individual coping strategies may depend on the situation, intensity, frequency, and individual circumstances.

Denial had a mean score of 4.01 ± 2.00, placing it above several of the less frequently reported coping strategies but below positive reframing, support-seeking, active coping, and planning. Denials represent an attempt to minimize or reject aspects of a difficult situation. Its moderate level of reporting suggests that nurses used a range of coping responses rather than relying exclusively on one approach. This finding reinforces the multidimensional nature of coping and supports the decision to examine the Brief COPE domains individually rather than assigning participants to a single coping category.

Most individual coping domains did not differ significantly according to gender, nationality, educational qualification, department, or years of professional experience after adjustment for multiple comparisons. This pattern suggests that the reported frequency of individual coping responses was broadly similar across the demographic and professional groups examined. However, the absence of statistically significant differences should not be interpreted as evidence that demographic or professional characteristics are unrelated to coping. The study was conducted within a single institution, and some subgroups, particularly nurses working in the emergency department and home medical care, were relatively small, which may have limited the ability to detect subgroup differences.

In the unadjusted analysis, self-blame differed significantly according to nationality. Saudi nurses reported a higher mean self-blame score than non-Saudi nurses (3.65 ± 1.85 versus 2.20 ± 2.05; unadjusted *p* = 0.001). This was the strongest nominally significant difference identified in the subgroup analyses. However, after Benjamini–Hochberg false-discovery-rate correction for multiple comparisons, the association was no longer statistically significant (adjusted *p* = 0.065). This distinction is important when interpreting the findings. The unadjusted result may suggest a potential difference worthy of further investigation, but the adjusted analysis does not provide sufficient evidence to conclude that nationality is significantly associated with self-blame in this study’s population. Possible explanations involving cultural expectations, professional experiences, perceived responsibilities, or other contextual factors remain speculative because these factors were not directly assessed. Future research involving larger and more diverse samples could investigate whether cultural and workplace factors contribute to differences in self-blame among psychiatric nurses.

Venting also showed a statistically significant difference according to years of professional experience in the unadjusted analysis (*p* = 0.013). Nurses with less than five years of experience had the highest mean venting score (4.24 ± 1.74), followed by nurses with 5–9 years (4.18 ± 1.82), 10–15 years (3.60 ± 1.46), and more than 15 years of experience (3.21 ± 1.83). The descriptive pattern showed lower reported venting scores among nurses with greater professional experience; however, this pattern was not statistically significant after FDR adjustment and should therefore be interpreted cautiously. However, after Benjamini–Hochberg adjustment, the association was no longer statistically significant (adjusted *p* = 0.423). Therefore, the present findings do not provide sufficient evidence to conclude that professional experience is associated with venting. Nevertheless, the observed pattern may warrant further investigation. Early-career nurses may have different opportunities or preferences for expressing difficult emotions than nurses with longer professional experience, while experienced nurses may have developed a broader repertoire of responses through professional exposure. These possibilities cannot be confirmed by the current cross-sectional data and should be tested in future longitudinal or qualitative studies.

The findings can be interpreted within Lazarus and Folkman’s Transactional Model of Stress and Coping [10,18]. The model conceptualizes coping as a dynamic process involving cognitive and behavioral efforts to manage demands perceived as challenging or exceeding available resources. The relatively frequent reporting of positive reframing, active coping, planning, and social-support strategies is consistent with the model’s recognition that individuals may use multiple cognitive, behavioral, and interpersonal responses when dealing with demanding situations. Importantly, the present study assessed coping strategies rather than measuring the level of occupational stress itself. Occupational stress provides the contextual background for the study, but no independent stress scale or stress score was used in the analyses reported here. Consequently, the findings cannot establish whether psychiatric nurses experienced high or low levels of occupational stress, nor can they determine whether any particular coping strategy reduced stress or improved psychological well-being. This distinction is important when interpreting the findings and avoids attributing causal or outcome-related effects to the coping strategies observed.

### Strengths and Limitations

A strength of this study is the use of the Brief COPE, which allowed multiple coping responses to be examined individually rather than imposing a single coping category on participants. The study also included psychiatric nurses from different clinical units and examined coping across several demographic and professional characteristics. Furthermore, the application of the Benjamini–Hochberg false-discovery-rate procedure provides a more conservative interpretation of the multiple subgroup comparisons and reduces the likelihood of overinterpreting isolated nominally significant findings.

Several limitations should be considered. First, cross-sectional design limits the ability to assess changes in coping over time and precludes conclusions about temporal direction or causality. Second, coping strategies were assessed using self-reported responses and may therefore be subject to recall and social-desirability bias. Third, the study was conducted in a single mental health institution in Riyadh, which may limit the generalizability of the findings to psychiatric nurses working in other institutions or regions of Saudi Arabia and to psychiatric nursing settings internationally. Fourth, some clinical-unit subgroups were relatively small, which may have limited the statistical power to detect subgroup differences. Fifth, the substance-use domain of the Brief COPE could not be analyzed because neither of its two items yielded a valid response in the study sample; therefore, this aspect of coping could not be evaluated. Sixth, cultural factors may have influenced how participants interpreted and reported coping behaviors, particularly strategies such as religious coping and self-blame, but these cultural influences were not directly measured. Finally, potentially relevant organizational factors, such as staffing levels, workload, leadership support, workplace violence exposure, and organizational resources, were not assessed and may have contributed to differences in reported coping. The study assessed the frequency of reported coping strategies and did not establish their effectiveness or their relationships with burnout, psychological well-being, job satisfaction, patient outcomes, or other professional outcomes.

## 5. Conclusions

Overall, psychiatric nurses in this study most frequently reported positive reframing, instrumental support, active coping, planning, emotional support, and religious coping, whereas behavioral disengagement and humor were less frequently reported. Most individual coping strategies did not differ significantly according to the demographic and professional characteristics examined. Although self-blame differed by nationality and venting differed by years of experience in the unadjusted analyses, neither association remained statistically significant after false-discovery-rate adjustment. These findings provide a descriptive overview of coping patterns among psychiatric nurses in a mental health institution in Riyadh. They do not establish that coping strategies reduce occupational stress or improve psychological or professional outcomes. Nevertheless, the findings may help inform future research and the development of supportive initiatives aimed at strengthening nurses’ coping resources and professional well-being.

Future studies should recruit psychiatric nurses from multiple institutions and regions of Saudi Arabia to improve the generalizability of findings. Longitudinal research could examine whether coping patterns change with professional experience or changes in clinical responsibilities. Qualitative studies may also help explain why particular coping strategies, such as positive reframing, religious coping, or self-blame, are preferred by some nurses. In addition, future research could examine coping alongside objectively or independently measured occupational stress, burnout, psychological well-being, job satisfaction, resilience, and organizational support. Such studies would provide a more comprehensive understanding of how psychiatric nurses respond to the demands of mental health nursing and whether specific coping approaches are associated with meaningful occupational and psychological outcomes.

### Implications for Healthcare Practice, Management, and Policy

The findings have potential implications for psychiatric nursing practice, healthcare management, and workforce policy. The frequent reporting of positive reframing, active coping, planning, and instrumental and emotional support identifies coping responses that may be relevant when considering staff-support initiatives. However, the findings should not be interpreted as evidence that these strategies are inherently more effective or that individual nurses are solely responsible for managing occupational demands.

At the individual and team levels, psychiatric nursing services may consider opportunities for reflective practice, peer support, professional mentorship, and access to confidential psychological support. Mentorship may be particularly relevant for nurses early in their careers, given the descriptive pattern of greater venting among nurses with less professional experience, although this association was not statistically significant after multiple-comparison adjustment. At the unit and organizational levels, the findings support consideration of psychologically safe mechanisms through which nurses can discuss difficult clinical experiences, including critical incidents involving aggression, violence, suicide risk, or patient deterioration. Healthcare organizations may also consider workload and staffing assessments, manager education regarding occupational distress, workplace-violence prevention, and routine monitoring of staff well-being. These approaches recognize that coping occurs within an organizational environment and should not be framed solely as an individual’s responsibility. At the policy level, psychiatric workforce well-being may be considered as part of healthcare-system resilience and patient-safety planning. Organizational policies that support adequate staffing, safe working environments, access to confidential employee support services, and structured professional development may provide a broader framework for supporting psychiatric nursing staff. These recommendations are implications suggested by the observed coping patterns rather than interventions demonstrated to be effective by this cross-sectional study. Future longitudinal and intervention research is needed to determine whether specific individual, team, or organizational approaches improve nurses’ well-being or other professional outcomes.

## Figures and Tables

**Table 1:** Characteristics of the participant psychiatric nurses (*n* = 225).

Characteristics	*N*	%
**Nationality**		
Saudi	171	76.0%
Non-Saudi	54	24.0%
**Gender**		
Male	121	53.8%
Female	104	46.2%
**Age categories**		
20–25 years	19	8.4%
26–30 years	43	19.1%
31–35 years	58	25.8%
36–40 years	66	29.3%
41–46 years	24	10.7%
>46 years	15	6.7%
**Qualification**		
Nursing diploma	59	26.3%
Bachelor	145	64.4%
Postgraduate	21	9.3%
**Years of experience**		
<5 years	60	26.7%
6–10 years	65	28.9%
11–15 years	70	31.1%
>15 years	30	13.3%
**Department/unit**		
Inpatient	171	76.0%
Outpatient	31	13.8%
Emergency department	7	3.1%
Home medical care	16	7.1%

**Table 2:** Response of the nurses on the items reflecting their coping strategies in response to occupational demands.

Domains and Items of Coping	Mean	SD
1. Active coping	4.62	1.546
I’ve been concentrating my efforts on doing something about the situation I’m in.	2.34	0.826
I’ve been acting to try to make the situation better.	2.28	0.842
2. Planning	4.62	1.540
I’ve been trying to come up with a strategy about what to do.	2.34	0.825
I’ve been thinking hard about what steps to take.	2.28	0.837
3. Positive reframing	5.02	1.206
I’ve been trying to see it in a different light, to make it seem more positive.	2.41	0.663
I’ve been looking for something good in what is happening.	2.61	0.625
4. Acceptance	3.41	1.808
I’ve been accepting the reality of the fact that it has happened.	1.41	0.996
I’ve been learning to live with it.	2.00	0.961
5. Humor	2.52	1.303
I’ve been making jokes about it.	1.16	0.678
I’ve been making fun of the situation.	1.36	0.686
6 Religion.	4.47	1.544
I’ve been trying to find comfort in my religion or spiritual beliefs.	2.10	0.823
I’ve been praying or meditating.	2.37	0.786
7. Use of emotional support	4.56	1.677
I’ve been getting emotional support from others.	2.28	0.842
I’ve been getting comfort and understanding from someone.	2.28	0.837
8. Use of instrumental support	4.68	1.649
I’ve been getting help and advice from other people.	2.34	0.825
I’ve been trying to get advice or help from other people about what to do.	2.34	0.826
9. Self-distraction	4.34	1.606
I’ve been turning to work or other activities to take my mind off things.	2.08	0.85
I’ve been doing something to think about it less, such as going to movies, watching TV, reading, daydreaming, sleeping, or shopping.	2.26	0.805
10. Denial	4.01	1.997
I’ve been saying to myself, ‘This isn’t real.	2.00	1.001
I’ve been refusing to believe that it has happened.	2.01	1.002
11. Venting	3.80	1.696
I’ve been saying things to let my unpleasant feelings escape.	1.83	0.931
I’ve been expressing my negative feelings.	1.97	0.845
12. Behavioral disengagement	2.62	1.692
I’ve been giving up trying to deal with it.	1.16	0.912
I’ve been giving up the attempt to cope.	1.46	0.860
13. Self-blame	3.30	1.994
I’ve been criticizing myself.	1.71	1.057
I’ve been blaming myself for things that happened.	1.59	1.095

**Note:** Each Brief COPE domain consists of two items scored from 0 to 3, giving a possible domain score of 0–6. The substance-use domain was excluded because both items received no valid responses in the study sample.

**Table 3:** Differences in coping strategy scores according to nurses’ characteristics.

Characteristic	Group	Active Coping	Planning	Positive Reframing	Acceptance	Humor	Religion	Emotional Support	Instrumental Support	Self-Distraction	Denial	Venting	Behavioral Disengagement	Self-Blame
**Gender**	Male	4.50 ± 1.66	4.50 ± 1.66	5.07 ± 1.10	3.31 ± 1.72	2.40 ± 1.31	4.37 ± 1.59	4.46 ± 1.80	4.55 ± 1.77	4.29 ± 1.67	3.93 ± 1.73	3.63 ± 1.61	2.64 ± 1.63	3.51 ± 1.79
	Female	4.75 ± 1.40	4.74 ± 1.39	4.95 ± 1.32	3.54 ± 1.91	2.66 ± 1.28	4.58 ± 1.49	4.65 ± 1.52	4.84 ± 1.49	4.38 ± 1.54	4.11 ± 2.27	4.00 ± 1.77	2.59 ± 1.77	3.06 ± 2.19
	*p*	0.235	0.252	0.449	0.337	0.126	0.322	0.395	0.187	0.688	0.521	0.101	0.798	0.088
**Nationality**	Saudi	4.57 ± 1.50	4.57 ± 1.49	4.94 ± 1.23	3.55 ± 1.75	2.54 ± 1.32	4.50 ± 1.54	4.47 ± 1.66	4.67 ± 1.58	4.32 ± 1.62	4.04 ± 1.87	3.75 ± 1.66	2.64 ± 1.68	3.65 ± 1.85
	Non-Saudi	4.76 ± 1.70	4.76 ± 1.70	5.26 ± 1.10	2.98 ± 1.94	2.44 ± 1.25	4.35 ± 1.56	4.81 ± 1.72	4.70 ± 1.87	4.37 ± 1.58	3.93 ± 2.36	3.94 ± 1.82	2.54 ± 1.73	2.20 ± 2.05
	*p* (unadjusted)	0.442	0.426	0.092	0.054	0.626	0.532	0.186	0.904	0.746	0.713	0.474	0.688	**0.001**
	FDR-adjusted *p*	0.880	0.880	0.880	0.880	0.880	0.880	0.880	0.925	0.880	0.880	0.880	0.880	**0.065**
**Qualification**	Nursing diploma	4.53 ± 1.71	4.53 ± 1.71	5.19 ± 1.09	3.64 ± 1.82	2.49 ± 1.44	4.39 ± 1.61	4.44 ± 1.82	4.61 ± 1.75	4.34 ± 1.75	4.51 ± 1.84	3.61 ± 1.66	2.85 ± 1.69	3.58 ± 1.93
	Bachelor	4.63 ± 1.54	4.63 ± 1.53	4.96 ± 1.24	3.30 ± 1.82	2.57 ± 1.26	4.48 ± 1.53	4.55 ± 1.68	4.71 ± 1.61	4.34 ± 1.59	3.74 ± 2.03	3.87 ± 1.72	2.45 ± 1.70	3.17 ± 1.98
	Postgraduate	4.76 ± 1.14	4.76 ± 1.14	4.95 ± 1.32	3.52 ± 1.69	2.29 ± 1.23	4.62 ± 1.50	4.86 ± 1.20	4.67 ± 1.71	4.24 ± 1.34	4.48 ± 1.89	3.86 ± 1.68	3.14 ± 1.49	3.48 ± 2.25
	*p*	0.816	0.820	0.459	0.457	0.645	0.838	0.622	0.925	0.960	0.054	0.608	0.102	0.378
**Department**	Inpatient	4.61 ± 1.60	4.60 ± 1.60	5.02 ± 1.20	3.39 ± 1.83	2.49 ± 1.33	4.42 ± 1.60	4.53 ± 1.76	4.67 ± 1.68	4.36 ± 1.60	3.99 ± 1.91	3.81 ± 1.67	2.56 ± 1.62	3.36 ± 1.91
	Outpatient	4.58 ± 1.46	4.58 ± 1.46	5.16 ± 1.13	3.48 ± 1.88	2.61 ± 1.28	4.71 ± 1.37	4.58 ± 1.48	4.58 ± 1.57	4.03 ± 1.92	3.74 ± 2.29	3.42 ± 1.88	2.71 ± 2.05	2.61 ± 2.36
	Emergency department	4.43 ± 1.72	4.57 ± 1.62	4.71 ± 1.50	3.14 ± 0.69	2.71 ± 0.95	3.86 ± 1.07	4.43 ± 1.62	4.57 ± 1.90	5.00 ± 1.29	5.00 ± 1.53	3.57 ± 1.13	2.86 ± 1.35	4.86 ± 0.90
	Home care	4.88 ± 1.09	4.88 ± 1.09	4.88 ± 1.41	3.69 ± 1.85	2.63 ± 1.31	4.75 ± 1.44	4.75 ± 1.24	5.00 ± 1.46	4.38 ± 1.03	4.38 ± 2.45	4.50 ± 1.67	3.00 ± 1.90	3.38 ± 2.16
	*p*	0.904	0.920	0.778	0.895	0.912	0.469	0.863	0.507	0.420	0.219	0.737	0.111	0.267
**Years of experience**	<5 years	4.76 ± 1.30	4.76 ± 1.30	5.18 ± 1.00	3.56 ± 2.02	2.38 ± 1.02	4.41 ± 1.54	4.65 ± 1.54	4.88 ± 1.41	4.12 ± 1.41	3.65 ± 2.12	4.24 ± 1.74	2.29 ± 2.07	3.44 ± 2.06
	5–9 years	4.74 ± 1.43	4.74 ± 1.43	5.18 ± 1.11	3.37 ± 1.76	2.48 ± 1.29	4.63 ± 1.56	4.68 ± 1.45	4.81 ± 1.47	4.40 ± 1.88	3.77 ± 2.05	4.18 ± 1.82	2.73 ± 1.64	3.44 ± 1.96
	10–15 years	4.43 ± 1.72	4.42 ± 1.70	4.90 ± 1.29	3.42 ± 1.77	2.47 ± 1.25	4.31 ± 1.57	4.33 ± 1.90	4.51 ± 1.86	4.36 ± 1.49	4.18 ± 1.90	3.60 ± 1.46	2.61 ± 1.63	3.39 ± 1.92
	>15 years	4.79 ± 1.47	4.79 ± 1.47	4.91 ± 1.31	3.33 ± 1.87	2.88 ± 1.69	4.67 ± 1.47	4.85 ± 1.50	4.73 ± 1.57	4.33 ± 1.63	4.36 ± 2.03	3.21 ± 1.83	2.76 ± 1.56	2.67 ± 2.16
	*p* (unadjusted)	0.466	0.434	0.406	0.957	0.382	0.527	0.375	0.592	0.859	0.298	**0.013**	0.635	0.267
	FDR-adjusted *p*	0.880	0.880	0.880	0.960	0.880	0.887	0.880	0.880	0.954	0.880	**0.423**	0.932	0.880

**Note:** Values are presented as mean ± standard deviation. *p*-values were obtained using independent-samples *t*-tests for two-group comparisons and one-way ANOVA for comparisons involving three or more groups. Because multiple comparisons were performed across 13 coping domains and five nurse characteristics, Benjamini–Hochberg false-discovery-rate (FDR) correction was applied. The FDR-adjusted *p*-values are presented for the two comparisons with nominal *p* < 0.05; neither remained statistically significant after correction. Bold value = significance.

## Data Availability

The raw data supporting the conclusions of this article will be made available by the authors on request.
